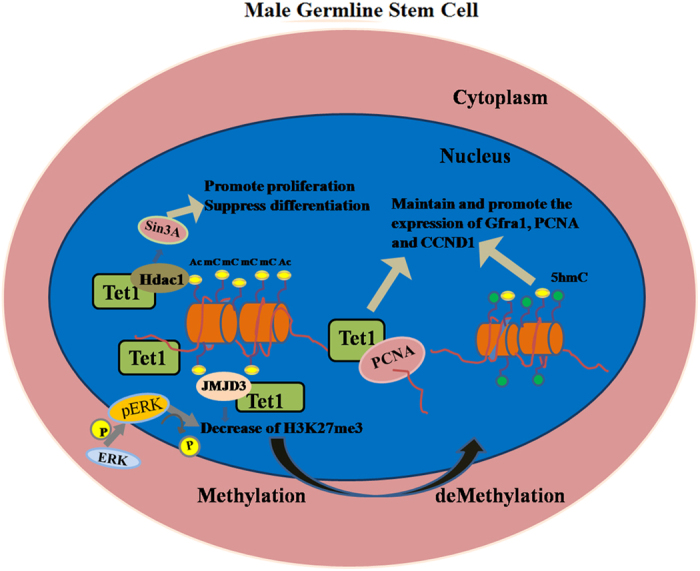# The Modification of Tet1 in Male Germline Stem Cells and Interact with PCNA, HDAC1 to promote their Self-renewal and Proliferation

**DOI:** 10.1038/srep37414

**Published:** 2016-11-18

**Authors:** Liming Zheng, Yuanxin Zhai, Na Li, Fanglin Ma, Haijing Zhu, Xiaomin Du, Guangpeng Li, Jinlian Hua

**Affiliations:** 1 College of Veterinary Medicine, Shaanxi Centre of Stem Cells Engineering & Technology, Northwest A&F University, Yangling, Shaanxi, 712100 China; 2 Key Laboratory for Mammalian Reproductive Biology and Biotechnology, Ministry of Education, Inner Mongolia University, Hohhot, 010021, China

## Abstract

Epigenetic modification plays key roles in spermatogenesis, especially DNA methylation dynamic is important in sustaining normal spermatogenesis. Ten-eleven translocation 1 (Tet1) is not only a key demethylase, which works in specific gene regions, but also crosstalks with partners to regulate epigenetic progress as protein complexes. Dairy goat is an important livestock in China, while the unstable culture system *in vitro* inhibits optimization of new dairy goat species. The study of epigenetic modification in male germline stem cells (mGSCs) is beneficial to the optimization of adult stem cell culture system *in vitro*, and the improvement of sperm quality and breeding of selected livestock. In our study, we not only analyzed the morphology, gene expression, DNA methylation and histone methylation dynamic in mouse Tet1 (mTet1) modified mGSCs, we also analyzed the stemness ability by *in vivo* transplantation and explored the functional mechanism of Tet1 in dairy goat mGSCs. The results showed mTet1 modified mGSCs had better self-renewal and proliferation ability than wild-type mGSCs, mTet1 could also up-regulate JMJD3 to decrease H3K27me3, which also showed to suppress the MEK-ERK pathway. Furthermore, Co-IP analysis demonstrated that TET1 interact with PCNA and HDAC1 by forming protein complexes to comprehensively regulate dairy goat mGSCs and spermatogenesis.

Epigenetic modification on DNA and histone level plays important roles in the development of embryonic and mGSCs through gene regulation, genomic imprinting, X-chromosome inactivation and carcinogenesis^1^,^2^. Tet1 was firstly found in acute myeloid leukemia (AML)^3^, the further study in ESCs showed Tet1 as a specific DNA demethylase for active DNA demethylation, could act as oxidant and turn the fifth position of cytosine (5mC) to 5-Hydroxymethylcytosine (5hmC) and the subsequent derivatives 5-formylcytosine (5fC) and 5-carboxylcytosine (5caC)^4^. Previous studies have elucidated the functions of Tet1 in mouse ESCs^5^,^6^, neuronal cells^7^, human ESCs^8^. Tet1 could take place of Oct4 and stimulate somatic cell reprogramming with other transcriptional factors such as Sox2, Klf4 and c-Myc^9^. While the development of primordial germ cells (PGCs), Tet1 is required for proper erasure of genomic imprints^10^, germ cell cancers utilize the oxidative pathway to achieve active DNA demethylation^11^. In recent years, more and deeper studies via model mice on Tet1 showed Tet1 functions in many biological processes^10^,^12^,^13^,^14^. Except for DNA demethylation^15^, Tet1 also works in various ways and crosstalks with partners to regulate gene expression and cancer^16^,^17^,^18^,^19^,^20^,^21^. Vitamin C (Vc) is important in Tet1 activity^5^,^22^, calpain could mediate TET1 degradation^23^, and promoted glycosylation of chromatin by binding to O-N acetyl glucose transferase (OGT) and mediate posttranscriptional modification^24^,^25^.

Mammalian spermatogenesis is a precise balance between the self-renewal of spermatogonia stem cells (SSCs) and their differentiation into spermatocyte and spermatid. The mGSCs have exhibited great pluripotency and ability for self-renewal and differentiation, during this devwloping progress, S6 ribosomal protein showed important roles in cell proliferation and network regulation through phosphorylation^26^,^27^,^28^. Previous study showed that DNA methylation patterns during mammalian spermatogenesis are changing^2^, the successfully migrated SSCs maintain self-renewal and differentiation by methylation dynamic in adult life cycle^2^. Abnormal DNA methylation may lead to male mouse sterility^29^,^30^. Tet1 defective mouse showed lower birth weight in their offspring^10^. Tet1 also showed higher expression level in diploid cells than haploid cells^2^, which evidenced Tet1 is essential in spermatogenesis and involved in epigenetic modification process during spermatogenesis^2^,^31^. As a key part of epigenetic modification, histone modification plays important roles in spermatogenesis. As a histone deacetylase, Sin3A, a member of the Sin3 family, which is linked to tumorigenesis and regulate gene expression through their role as histone deacetylases (HDACs)^32^, it was also distinctly required in differentiating spermatogonia and cell cycle progression^33^,^34^. Another histone deacetylase we noted is Hdac1, which could cooperate with Sin3A as SIN3A-HDAC1 complex, Hdac1 could also protect the testicular damage^35^,^36^. Histone modification also functions in both chromatin structure repression and activation, such as lysine 9 and 27 on histone H3 for gene repression and lysine, 36, and 79 on histone H3 for gene activation when they are methylated^37^,^38^. The different methylation patterns of K3K4me3 and H3K27me3 consisted Tet1 showed a special role in DNA demethylation mediated by Tet1^39^.

Up to now, study on dairy goat mGSCs is hard to get deeper because it remains difficult to establish stable dairy goat mGSC cell lines and *in vitro* culture systems^40^,^41^. We optimized *in vitro* culture systems for dairy goat mGSCs and have successfully established an immortalized dairy goat mGSC cell line^42^, enabling the long-term study of dairy goat mGSCs *in vitro*^42^,^43^,^44^. In order to maintain pluripotency and reprogramming efficiency of dairy goat mGSCs, we investigate the mGSC biology through Tet1 overexpression, verified the self-renewal and proliferation ability *in vivo* and *in vitro* to explore the role of Tet1 in mGSC characters and development potentiality.

## Results

### Generation and Identification of Transgenic mGSCs with mGSC-pCDH-mTet1 and mGSC-pCDH

**Figure 1:**
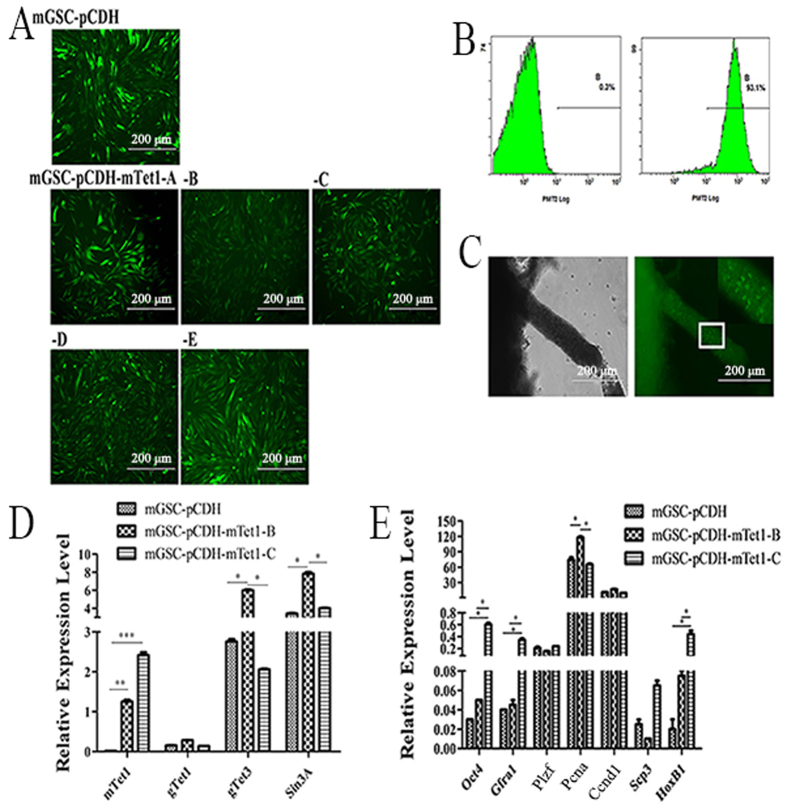
The positive cell clone screen and gene expression of mGSC-pCDH and mGSC-pCDH-mTet1 cells. (**A**) The mGSCs were transfected with pCDH-mTet1 and screened positive clonal cells (mGSC-pCDH-mTet1-A-E with different GFP level). Bar = 200 μm. (**B**) The FACS assay analyzes the purity of mGSC-pCDH-mTet1 cells. (**C**) The mice seminiferous tubule with mTet1 overexpressed lentivirus for 2 days. Bar = 200 μm. (**D**) Gene expression (mTet1, gTet1, gTet3, Sin3A) of mGSC-pCDH and mGSC-pCDH-mTet1 cells analyzed by QRT-PCR. **p < 0.01, *p < 0.05. (**E**) Gene expression (Oct4, PLZF, Gfra1, PCNA, CCND1, Scp3 and HoxB1) of mGSC-pCDH and mGSC-pCDH-mTet1 cells analyzed by QRT-PCR. **p < 0.01, *p < 0.05.

The homology comparison of Tet1 conserved domains among cattle, goat, human and mouse showed they share the same key functional domains. So mTet1 was transfected into dairy goat mGSCs to detect the role of Tet1 in mGSCs (Table S1–3). The fluorescence reporter green fluorescent protein (GFP) was constructed to monitor mTet1 expression in living cells. The mTet1 modified mGSCs clone was screened with puromycin at 500 μg/ml, while mGSCs with pCDH vector had lower tolerance at 250 μg/ml. The positive clones were identified by GFP expression and we screened 5 clones (mGSC-pCDH-mTet1-A to E) to analyze (Fig. 1A) and the clone purity was higher than 93% (Fig. 1B). The mouse seminiferous tubules were transduced with lentivirous and the expression of GFP showed the reconstructed lentivirous vector has infection ability (Fig. 1C). In different mTet1 positive cell clones, qRT-PCR analysis showed dynamic in Tet1 related genes and germline specific genes (Fig. 1C,D). Although mGSC-pCDH-mTet1-C clone showed higher expression of Oct4 (P < 0.05) and Gfra1 (P < 0.05), it also presented higher expression of Scp3 and HoxB1 (P < 0.05), which were related to cell differentiation. So, we chose mGSC-pCDH-mTet1-B clone, as it was stable not only in moderately strengthen of male germline cell characteristics, such as up-regulation of Oct4, Gfra1 and down-regulation of Scp3, but also increased the proliferation gene Pcna (P < 0.05) in mGSC-pCDH-mTet1 cells compared with mGSCs-pCDH, providing further evidences that Tet1 overexpression promotes mGSC proliferation (Fig. 1D). Besides, the up-regulation of gTet1 (P < 0.01), gTet3 (P < 0.05) and Sin3A (P < 0.05) in mGSC-pCDH-mTet1 cells showed mTet1 could activate endogenous Tet1 expression and authenticated target Sin3A. These results demonstrate that Tet1 plays an important role in the regulation of dairy goat mGSCs.

### The differentiation ability of mGSC-pCDH-mTet1 and mGSC-pCDH cells

**Figure 2:**
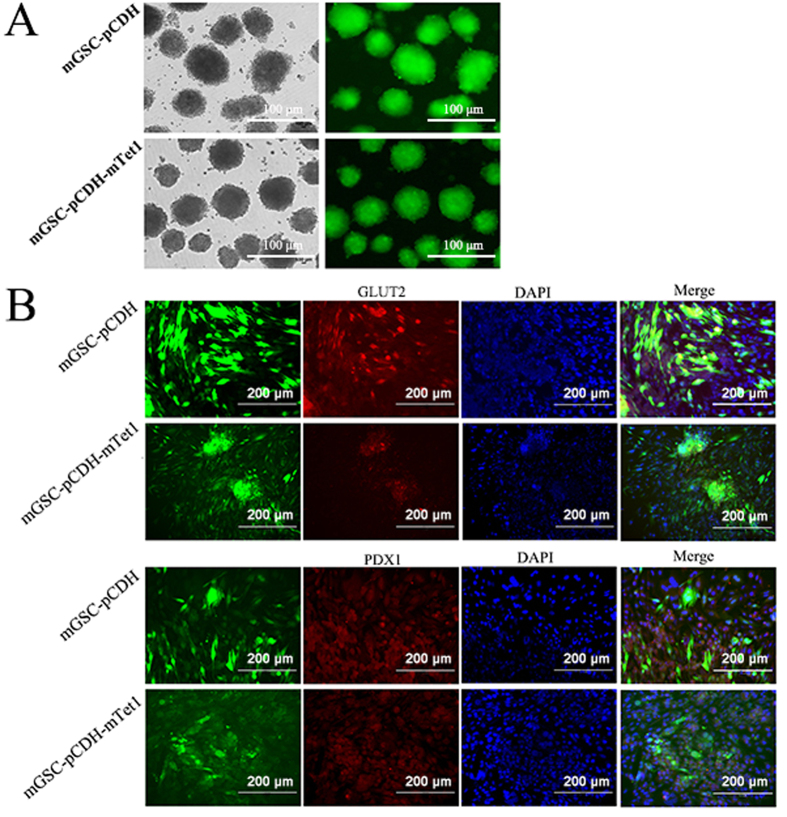
The differentiation ability of mGSC-pCDH and mGSC-pCDH-mTet1 cells *in vitro*. (**A**) The EB formation in mGSC-pCDH and mGSC-pCDH-mTet1 cells. Bar = 100 μm. (**B**) Immunofluorescence staining of three germ layers spontaneous differentiation markers (Glut2, Pdx1) in mGSC-pCDH and mGSC-pCDH-mTet1 cells. Bar = 200 μm.

The previous study showed mGSCs have differentiation ability *in vitro* and fertile offspring^45^. The EB formation assay showed mGSC-pCDH-mTet1 cells had better EB formation ability, which presented more EBs with more uniform diameter, smooth surface and density compared with mGSC-pCDH cells (Fig. 2A). Spontaneous differentiation of EBs showed the expression of three layer marker GLUT2 and PDX1 were lower in mGSC-pCDH-mTet1 cells than mGSC-pCDH cells (Fig. 2B) after 7 days culture, which exhibited a lower differentiation rate in the same culture condition with mTet1 modification compared with control. The results demonstrate that Tet1 played key roles in mGSC self-renewal and differentiation inhibition.

### The self-renewal ability of mGSC-pCDH and mGSC-pCDH-mTet1 cells

**Figure 3:**
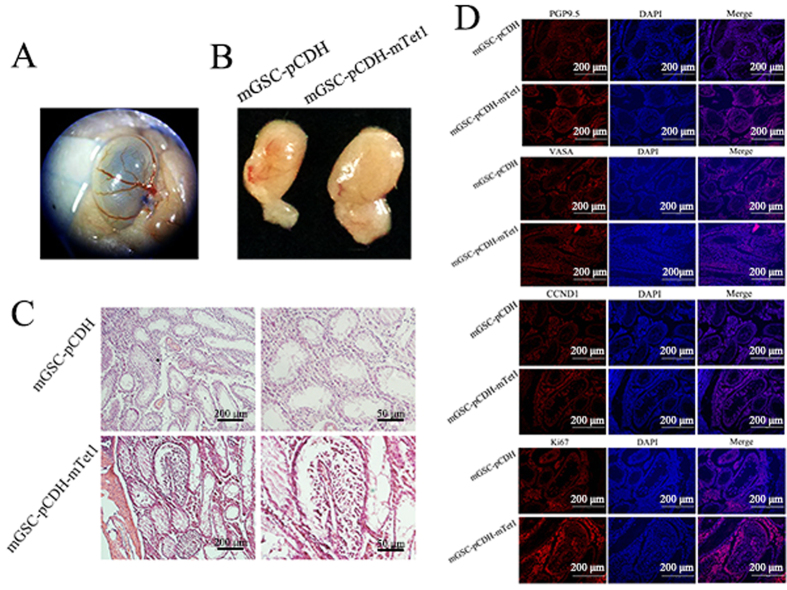
The self-renewal ability of mGSC-pCDH and mGSC-pCDH-mTet1 cells *in vivo*. (**A**) Transplantation of mGSC-pCDH and mGSC-pCDH-mTet1 cells into busulfan treated mice’s seminiferous tubules. (**B**) The busulfan treated mice seminiferous tubule with mGSC-pCDH and mGSC-pCDH-mTet1 cells for 30 days. (**C**) The HE staining of busulfan treated mice seminiferous tubule with mGSC-pCDH and mGSC-pCDH-mTet1 cells for 30 days, Bar = 200 μm for left two pictures, Bar = 50 μm for right two pictures. (**D**) Immunofluorescence staining of PGP9.5, VASA, CCND1 and Ki67 in busulfan treated mice seminiferous tubule with mGSC-pCDH and mGSC-pCDH-mTet1 cells. Bar = 200 μm.

The golden standard of mGSC biological characteristics is their capacity to form colonies in the transplantated recipient testis’ seminiferous tubule^46^,^47^. In order to identify the dairy goat mGSC characteristics and whether Tet1 effects on the fate of mGSCs, we transplantated mGSC-pCDH and mGSC-pCDH-mTet1 cells into infertility mouse testes treated with busulfan (Figs S1 and 3A). 8 male recipients received cell transplantation at 12 week of age, and all were killed after 4 weeks. When we examined the testes from 3 recipients, we observed in the same mouse, the testis transplantated with mGSC-pCDH-mTet1 cells showed bigger size than the other one with mGSC-pCDH cells (Fig. 3B), and HE staining showed there were more cells in the tubules of testis transplantated with mGSC-pCDH-mTet1 cells, while the control showed no cell proliferation (Fig. 3C). Moreover, the germline specific gene PGP9.5 and VASA, and proliferative gene CCND1 and KI67 were positive in the transplantated mGSCs (Fig. 3D), which verified that dairy goat mGSCs sustained self-renewal potential and Tet1 modified mGSCs showed stronger self-renewal and proliferation ability.

### Tet1 demethylation and effect on histone methylation

**Figure 4:**
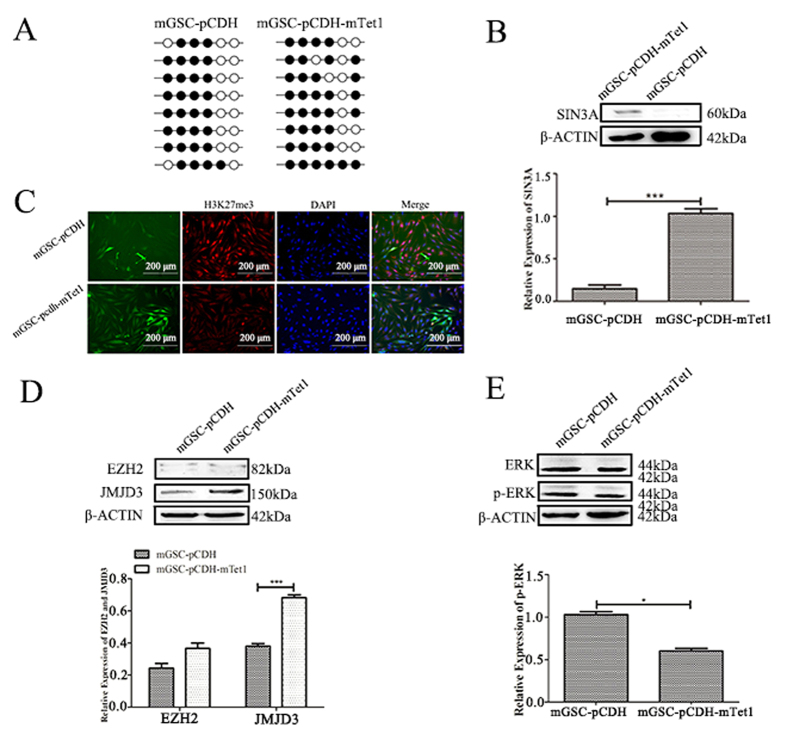
Tet1 modification as demethylase and its effect on histone methylation. The methylation level of Oct4 promoter in mGSC-pCDH and mGSC-pCDH-mTet1 cells. (**B**) Western blot analysis of mGSCs transfected with pIRES2-AcGFP-Sin3A. (**C**) Immunofluorescence staining of H3K27me3 in mGSC-pCDH and mGSC-pCDH-mTet1 cells. Bar = 200 μm. (**D**) Western blot analysis of Histone methylase EZH2 and histone demethylase JMJD3 of H3K27me3 in mGSC-pCDH and mGSC-pCDH-mTet1 cells. (**E**) Western blot analysis of ERK and p-ERK in mGSC-pCDH and mGSC-pCDH-mTet1 cells.

As a DNA demethylase, Tet1 plays key roles in specific genes by promoter demethylation in ESCs. In dairy goat mGSCs, we firstly demonstrated that Tet1 had no obviously regulation on Oct4 promoter, as the methylation level on Oct4 promoter showed no significant difference between mGSCs and mTet1 modified mGSCs (Fig. 4A), even though Oct4 was up-regulated in Tet1 modified mGSCs. Sin3A was a confirmed predicted target gene of Tet1 (Fig. 4B), and we verified its up-regulation in Tet1 modified mGSCs (P < 0.001), which showed mTet1 worked in dairy goat mGSCs, and indicated more regulatory ways of Tet1 in mGSCs. We have demonstrated that mGSC-pCDH-mTet1 cells showed lower H3K27me3 than mGSC-pCDH cells (Fig. 4C), the western blot showed histone demethylase JMJD3 presented higher expression in mGSC-pCDH-mTet1 cells than mGSC-pCDH cells (P < 0.001), in which the histone methylase EZH2 had no significant change (Fig. 4D). Besides, the level of pERK decreased in the same time (P < 0.05) (Fig. 4E). These results demonstrated that Tet1 could up-regulate JMJD3 to decrease H3K27me3, and suppress the MEK-ERK pathway in this process.

### Tet1 modification as protein complex with PCNA and Hdac1

**Figure 5:**
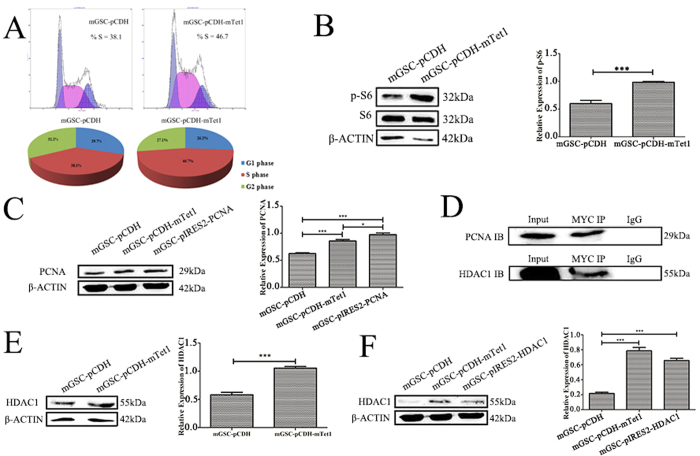
Tet1 modification as protein complex interaction with PCNA and Hdac1. (**A**) The FACS assay analysis the S phase dynamics of mGSC-pCDH and mGSC-pCDH-mTet1 cells. (**B**) Western blot analysis of S6 and pS6 in mGSC-pCDH and mGSC-pCDH-mTet1 cells. (**C**) Western blot analysis of PCNA in mGSCs transfected with pIRES2-AcGFP-PCNA. (**D**) Co-IP analysis of Myc (Tet1) with PCNA and Hdac1. (**E**) Western blot analysis of Hdac1 in mGSC-pCDH and mGSC-pCDH-mTet1 cells. (**F**) Western blot analysis of Hdac1 in mGSCs transfected with pIRES2-AcGFP-Hdac1.

**Figure 6:**
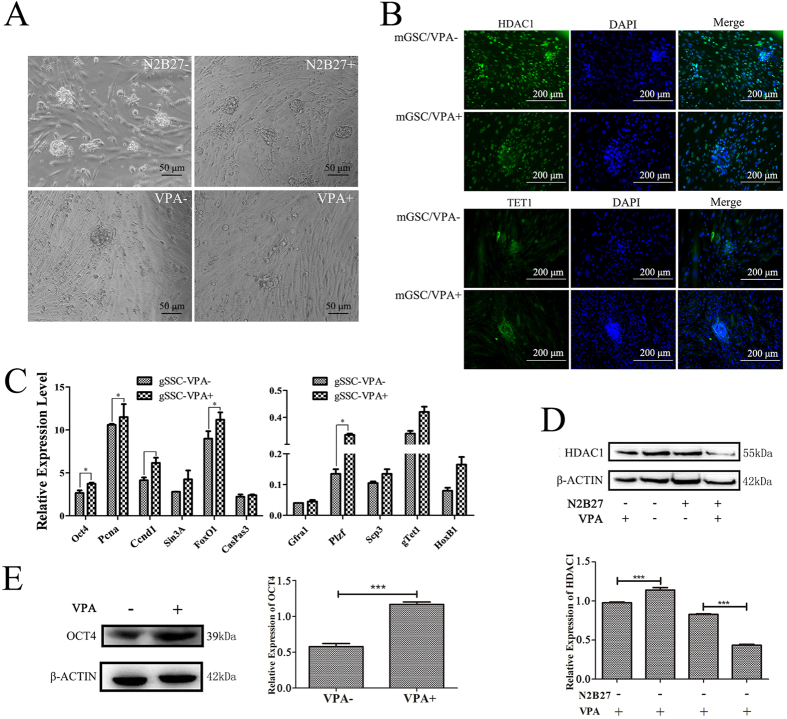
The regulatory mechanism of Hdac1 in mGSCs. (**A**) The morphologic study of mGSCs cultured with VPA and N2B27. Bar = 50 μm. (**B**) Immunofluorescence staining of Hdac1 and Tet1 in mGSCs treated with VPA. Bar = 200 μm. (**C**) Gene expression of mGSCs treated with VPA analyzed by QRT-PCR. n = 3, *p < 0.05. (**D**) Western blot analysis of Hdac1 in mGSCs cultured with VPA and N2B27. (**E**) Western blot analysis of Oct4 in mGSCs cultured with VPA.

In order to make further knowledge on Tet1 regulation way in mGSCs, we kept focusing on several key genes such as Pcna, Hdac1 and Sin3A. For Pcna, both the qRT-PCR and FACS analysis showed mGSC-pCDH-mTet1 cells had better proliferative ability than mGSC-pCDH cells (Figs 1e and 5a). The data of FACS showed there were more than 46% mGSC-pCDH-mTet1 cells in the S phase, while only 38% in mGSC-pCDH cells (Fig. 5A), the increasing of pS6 also proved Tet1 modified mGSCs had higher activity and vitality (P < 0.05) (Fig. 5B). So we choose Pcna as a predicted target gene to get overexpression in mGSCs transfected with pIRES2-Pcna, and western blot showed PCNA was up-regulated in mGSCs (P < 0.001), which had almost the same tendency as mGSC-pCDH-mTet1 cells compared with mGSC-pCDH cells (P < 0.05) (Fig. 5C). These results suggested TET1 interact with PCNA is a positive regulator to PCNA, the Co-IP assay showed TET1 form protein complex with PCNA and modify mGSC proliferation in protein level (Fig. 5D). The same validation was presented in another predicted target gene Hdac1, we have found that HDAC1 was up-regulated in mGSC-pCDH-mTet1 cells compared with mGSC-pCDH cells (P < 0.001) (Fig. 5E), then we got Hdac1 overexpression in mGSCs transfected with pIRES2-Hdac1, which was up-regulated in mGSCs (P < 0.001) (Fig. 5F), and the results of Co-IP evidenced that TET1 could also form protein complex with HDAC1 (Fig. 5D). As a histone deacetylase, the dynamic of HDAC1 affect the histone acetylation, which was an important component of epigenetic modification. In order to verify whether TET1 influence histone acetylation, mGSCs were treated with the inhibitor of HDAC1 sodium valproate (VPA), meanwhile, we added N2B27 to optimizate the mGSC culture system, and the mGSCs with N2B27 exhibited stronger clone formation ability and morphology, while the VPA addition declined the clone formation ability with smaller and incompact clones, which suggested HDAC1 have effects on mGSC self-renewal (Fig. 6A). On mRNA level, VPA treated mGSCs showed expression dynamic on mGSC specific markers, however not Tet1 and related genes. Oct4 and Plzf were significantly up-regulated, so as the Pcna, Ccnd1 and FoxO1, which were important in sustaining self-renewal of mGSCs (Fig. 6C). The protein level of Oct4 also verified these results (Fig. 6E). Besides, the VPA treated mGSCs also showed obviously decrease of HDAC1 on protein level (Fig. 6B,D), while TET1 showed no change, just was corresponding with expression in mRNA level, which suggested that Hdac1 is not an upstream target gene of Tet1. TET1 may play a role of recruitment with HDAC1 by forming protein complex to affect histone acetylation, which may further influence chromosome structure and gene transcription activation.

Taken together, the overexpression of mTet1 in goat mGSCs showed variances in cellular morphology, gene expression and epigenetic dynamic. For functional regulation, TET1 had novel and dynamic roles in sustaining self-renewal of mGSCs and keep proliferation by directly DNA demethylation or crosstalking with partner protein (Fig. 7), which may provide better methods for epigenetic modification mechanism of Tet1 in mGSCs.

## Discussion

The earlier research advancement about Tet1 was focusing on ESCs. The significant achievements on iPSCs also showed Tet1 could promote expression of Oct4 and reprogramming efficiency^12^, even replace Oct4 and activate somatic cells reprogramming^9^. Recent study of Tet1 were about cancer, 5hmC got lower level in patients with breast cancer, liver cancer, lung cancer, pancreatic cancer and gastric cancer^13^,^48^,^49^,^50^,^51^,^52^,^53^, which showed down-regulation of Tet1 may lead to cancer and Tet1 act as a tumor suppressor in epigenetic modification. Moreover, Tet1 functions in different cell types via various regulation pathways^10^,^12^,^13^,^14^. Our previous study described the expression patterns of Tet1, H3K9 and H3K27 in dairy goat testis and cultured goat spermatogonia stem cells (gSSCs)^54^, we further detected the expression of histone demethylase JMJD3 and histone methylase EZH2, and found that it is the up-regulation of JMJD3 lead to a decline of H3K27me3, which showed TET1 interact with JMJD3 to achieve demethylation. Meanwhile, histone methylase EZH2 had no significant expression dynamic, which revealed TET1 had no direct regulation with EZH2. As an oncogene, EZH2 presented high expression level in cancer cells through activation of MEK-ERK pathway to increase the amount of H3K27me3^55^. The low and stable level of EZH2 also demonstrated mTet1 modified dairy goat mGSCs did not concerate as oncogene and tumor suppressor are altering or replacing each other^56^, it kept normal with stable self-renewal and proliferative ability. Besides, under the premise of definite cell types, several mouse cell lines such as C2C12, C127 and NIH3T3 were treated with inhibitor of MEK-ERK pathway. In this process, EZH2 was down-regulated and JMJD3 was up-regulated, while another histone methylase EZH1 and histone demethylase UTX showed no change, which verified the dynamic of EZH2 and JMJD3 suppress MEK-ERK pathway in specific cell types^57^. In our study, we summarized an epigenetic regulation model of Tet1 and its interrelated pathway and interacting proteins in dairy goat mGSCs (Fig. 7). In dairy goat mGSCs, it is the TET1 and JMJD3 that affect activation of MEK-ERK pathway to support the self-renewal of dairy goat mGSCs.

As an important epigenetic modification enzyme, Tet1 interact with other proteins by forming protein complex^58^, transcription factor Lin28 regulate gene expression through recruitment of TET1^59^. An active DNA demethylation process occurs with an involvement of TET enzymes, and the expression level of TET1–3 is critical for human spermiogenesis and male fertility^31^. In our previous study, we found that mGSC-mTet1 cells proliferated at a significantly greater rate than wild-type mGSCs, and mGSCs-specific markers such as proliferating cell nuclear antigen (PCNA), cyclinD1 (CCND1), GDNF family receptor alpha 1 (Gfra1) and endogenic Tet1, Tet2 were up-regulated. Our results conclusively demonstrate that modification of mGSCs with mTet1 affected mGSC maintenance and seemed to promote establishment of stable goat mGSC cell lines^43^. Further, we used Co-IP analysis to demonstrate that TET1/PCNA complex is effective in the modulation of proliferation as a highly specific manner. Histone acetylation dynamic played important epigenetic regulatory roles in induced pluripotent stem cells (iPSCs) formation, previous study showed HDAC2 was antagonism with TET1 in the same gene regions, so TET1 promote iPSCs formation by decreasing the HDAC2 binding sites, and change histone acetylation of key genes^60^. In our study, Hdac1 was recruited specifically by mTET1 and formed protein complex to regulate histone acetylation, moreover, the complex bind to key genes and regulate gene and protein expression of mGSCs. Tet1 showed no significant change in mGSCs treated with VPA as an inhibitor of HDAC1, which suggested the expression of Tet1 was not the direct upstream target gene of Hdac1 and had not been affected. Besides, Oct4 showed up-regulation in mGSCs treated with VPA than without treatment, so the high level of Hdac1 may suppress expression of Oct4 and affect the maintainance of pluripotency. While the formation of TET1/HDAC1 complex evidenced obvious epigenetic modifications to maintain dairy goat mGSC self-renewal. Another predicted target gene skeleton protein Sin3A showed up-regulation in Tet1 modified mGSCs, there was ChIP-seq data of mESCs showed Tet1 crosstalk with histone acetylase Mof and Sin3A on chromosomal alterations by forming TET1/SIN3A/hMOF complex, which mediated acetylation of H4K16 and regulate gene expression related to DNA repair during DNA double strand break (DSB)^61^.

Taken together, our study showed the regulatory mode of Tet1 with specific genes and proteins, verified the modification mechanism of Tet1, which demonstrate the importance of Tet1 in mGSC self-renewal and provide new perspectives for epigenetic modifications mediated by Tet1 in dairy goat mGSCs.

## Conclusion

DNA methylation, Histone methylation and acetylation within certain stages are crucial during mammalian development. In our study, TET1 not only modulates DNA methylation, TET1 also participates in histone modification via binding to gene promoters or forming compound with other proteins to drive the dynamic of epigenetic modification in mGSCs. We found the dynamic of H3K27me3 were suggested to be associated with histone demethylase JMJD3. TET1 interact with PCNA and HDAC1 as complex to regulate proliferation and gene expression in dairy goat mGSCs. Our results demonstrate Tet1 present important roles in the maintenance of mGSC self-renewable stability and provide new perspectives for DNA methylation/demethylation and better regulation of epigenetic modifications in dairy goat mGSCs.

## Materials and Methods

### Ethics statement

All animal experiments were performed in strict accordance with the Guide for the Care and Use of Laboratory Animals (Ministry of Science and Technology of the People’s Republic of China, Policy No. 2006 398) and were approved by the Animal Care and Use Center of the Northwest A&F University.

### Plasmid construction

For plasmid construction, primers for gene clone were as Table S1-1, and the restriction enzyme cutting sites of vector pIRES2-AcGFP were as Table S1-2. To reconstruct plasmid pCDH-CMV-MCS-EF1-mTet1 (pCDH-mTet1), the vector pCDH-CMV-MCS-EF1 (pCDH) was digested with BamHI/NotI and made a junction between linearized vector and the 6120 bp mTet1 fragment generated from pTRE3G-BI-mTet1^43^.

### Cell Culture and DNA transfection

The mGSCs used in this study was an immortalized dairy goat mGSC line mGSCs-I-SB as our lab storage. They were cultured in DMEM/F12 (Invitrogen, Carlsbad, CA) supplemented with 10% fetal bovine serum (FBS, Hyclone, USA), 0.1 mM ß-mercaptoethanol (Sigma, St. Louis, MO) and 2 mM glutamine (Invitrogen) in a humidified atmosphere with 5% CO_2_ at 38.5 °C. The 293T cells were cultured as the conventional conditions.

For DNA transfection of mGSCs, Tuberfect was used as a transfection reagent, when cells were cultured with the density of 75%, fresh DMEM/F12 consisted of all the supplements were replaced. 30 min later, Tuberfect and plasmid were co-incubated with a volume V = (m of plasmids/2) at room temperature for 15 min, then added to and blending with the medium. 12 h later, fresh DMEM/F12 contained all the supplements were replaced. 24 h later, cells were diluted with puromycin (500 μg/ml) to screen pCDH-mTet1 cell clones and 250 μg/ml for pCDH cell clones. Single cell clones were isolated 8–12 days after dilution culture and expanded for further culture. It should be noticed that for 293T cells, before DNA transfection, fresh DMEM/F12 without FBS was replaced so that we had a higher transfection efficiency.

### Immunofluorescence

For slides of tissues, before fixation, they were firstly dewaxed and soaked in boiling citrate buffer for 15 to 25 min to get natural cooling. For the cell samples, they were directly fixed in 4% PFA in PBS for 15 min and 0.5% Triton X-100 in PBS for 10 min, blocked in 4% goat serum at room temperature for 30 min, and then exposed to primary antibody TET1 (1:500; Cell Signaling Tecnology, Inc., USA), GLUT2 (1:200; Bioss, Beijing, China), PDX1 (1:200; Bioss, Beijing, China), PGP9.5 (1:50; Bioss, Beijing, China), VASA (1:100; Sangon Biotech, Shanghai, China), CCND1 (1:100; BOSTER, Wuhan, China), KI67 (1:100; Bioss, Beijing, China), HDAC1 (1:100; Sangon Biotech, Shanghai, China) and H3K27me3 (1:200; Sino Biological Inc.) overnight at 4 °C. After three 5 min washes in PBS, samples were incubated with secondary Alexa 594 anti-rabbit/mouse IgG antibody (1:200; Invitrogen) at room temperature for 30 min, followed by three washes in PBS. Cell nuclei were stained using DAPI. Images were captured using a Leica fluorescence microscope (Hicksville, NY, USA)^43^.

### Immunofluorescence of 5hmC

For slides of tissues, before fixation, they were firstly dewaxed and soaked in boiling citrate buffer for 15 to 25 min to get natural cooling. For the cell samples, they were directly fixed in 4% PFA in PBS for 15 min, then 0.5% Triton X-100 in PBS for 10 min. After three washes in PBS, the slides or cell samples were incubated with RNase (50 μg/ml) at 37 °C for 1 h in the dark, 4 N HCl at 37 °C for 20 min, and then 100 mM Tris-HCl at room temperature for 10 min. After three washes in PBS, samples were blocked in 4% goat serum at room temperature for 30 min and exposed to a primary antibody against 5hmC (1:500; Active Motif) at 4 °C overnight. Then, after three 5 min washes in PBS, samples were incubated with Alexa 594 anti-rabbit IgG antibody (1:500; Invitrogen) at room temperature for 30 min followed by three washes in PBS. The cell nuclei were stained using PI (Prodium Iodide). Images were captured using a Leica fluorescence microscope (Hicksville, NY, USA)^43^,^54^.

### Quantitative RT-PCR (qRT-PCR)

Total RNA was extracted from mGSCs and mGSC-mTet1 cells using TRIzol (Tiangen Biotech Co. Ltd, Beijing, China). Primers were designed using published sequences and listed in Table S2. Detailed qRT-PCR and gel electrophoresis protocols can be found in previous studies^54^. The expression levels of genes were analyzed by a quantitative reverse transcriptase-polymerase chain reaction (qRT-PCR) as described previously^62^.

### Western blot assay

Proteins were extracted from stably transfected cells and then the protein concentration was detected using the BCA Protein Quantification Kit (Vazyme, Piscataway, NJ, USA). After heat denaturation in 5% SDS–PAGE sample loading buffer, the protein samples were resolved by SDS-PAGE and transferred to a PVDF membrane. The samples were probed with β-ACTIN (1:1000; Sino Biological Inc., Beijing, China), S6/pS6 (1:1000; Cell Signaling Tecnology, Inc., USA), EZH2 (1:500; Sangon Biotech, Shanghai, China), JMJD3 (1:800; Abcam Inc., USA), ERK/pERK (1:500; Cell Signaling Tecnology), PCNA (1:500; BOSTER, Wuhan, China), SIN3A (1:300; Bioss, Beijing, China), HDAC1 (1:500; Sangon Biotech, Shanghai, China), OCT4 (1:500; Santa Cruz, Inc., USA) and H3K27me3 (1:1000; Sino Biological Inc., Beijing, China) as previously described^54^. The secondary antibody was horseradish peroxidase-conjugated anti-rabbit/mouse IgG (1:1,000, Bioworld, Nanjing, China). The detection was performed using Thermo Scientific Pierce ECL western blotting substrate, and the results were analyzed using a Tanon-410 automatic gel imaging system.

### Co-immunoprecipitation assay

For co-immunoprecipitation (Co-IP) assay, pCMV-Myc-mTet1 was cotransfected with pIRES2-AcGFP-PCNA or pIRES2-AcGFP-Hdac1 into 293T cells. 48 h after transfection, cells were harvested and washed with chilled PBS three times, then lysed in IP Lysis Buffer (Pierce, USA) with a Protease Inhibitor Cocktail (Sigma, USA). The protein was quantified by using a BCA Protein Assay kit (Beyotime, China), then diluted into 1 mg/ml with chilled wash buffer (with protease inhibitors). Took out 10 μl as Input, stored at −20 °C for use. Meanwhile, the rest protein was divided into two equal parts, which were added with the mouse anti-Myc antibody (Abbkine, USA) (1–2 μg) and the same amount of mouse IgG (Beyotime, China) respectively, rotating slowly, and incubated overnight at 4 °C. Protein A agarose beads were washed twice with cold wash buffer followed by adding them into the two parts of protein based on 20 μl every 1 mg total protein, then incubated for 4–6 hours at 4 °C with low-speed rotation. Afterwards, the protein mixture was washed with 500 μl chilled wash buffer (containing a protease inhibitor) for three times. Dilute all the protein mixtures, including the Input protein, with wash buffer. After heat denaturation in 5% SDS–PAGE sample loading buffer, the protein samples were subjected to western blot assay.

### Fluorescence Activated Cell Sorter (FACS) Assay

For the FACS-mediated cell cycle assay, the cells were digested by trypsin and washed in PBS at room temperature. After centrifugation, the PBS was discarded and 1 ml cold staining buffer was added. Cells were resuspended, and then 10 μl PI solution was added and incubated with the cells for 15 min in the dark. For the FACS-mediated cell purity assay, the cells were digested by trypsin and washed in PBS at room temperature, cells without GFP were used as negative control and set 0.1% as gating. FACS was performed on an EPICS ELITE apparatus (Beckmann-Coulter) with MultiCycle software (Phoenix Flow Systems, Inc.)^63^.

### Embryoid Body (EB) Formation

Cells were cultured at 38.5 °C in a humidified atmosphere with 5% CO2, and the cells were digested by trypsin with the cell density of 95%, then every 3 × 10^6^ cells were diluted in a 35-mm non-adhesive dish containing fresh DMEM-F12 with 10% FBS, so that cells were suspended growing and 3 days later, EBs formed.

### The mGSCs Transplantation

Transplantation was performed in the mouse testis with infertility induced by busulfan^64^, cells were delivered by cannulation connected to a pusher. Firstly, transplantation was performed under general anesthesia and aseptic surgical conditions. A linear incision was made lateral and parallel to the median raphe, and the testis enclosed in the parietal vaginal tunic was exposed. Then the efferent ductules of testis were found and isolated, and mGSCs-pCDH-mTet1 cells were delivered in seminiferous tubules by glass micropipette (Fig. S1). mGSCs-pCDH cells were delivered in another testis in the same way. The average volume of the cell suspension injected into each testis was 15–20 μl. All experiments were approved and performed in accordance with relevant guidelines and regulations of Northwest A&F University.

### Bisulfite Sequencing Assay

Genomic DNA was extracted from the modified mGSCs and subjected to sodium bisulfite treatment using the EZ DNA Methylation-Direct™ Kit (Zymo Research, USA) in accordance with the instruction manual. Each DNA sample was transferred to 20 μl digestion mixture. After incubation for 3 h at 50 °C, the digested samples were added to 130 μl CT Conversion Reagent for bisulfite conversion and incubated at 98 °C for 8 min and then at 64 °C for 3.5 h. Modified DNA was then desalted, purified, and finally eluted with 15 μl of elution buffer. Subsequently, Bisulfite Sequencing PCR (BS-PCR) was immediately carried out using 2 μl of modified DNA per PCR run. The primer of Oct4 promoter was listed in Table S1-1. The BS-PCRs were performed using the Hot Start DNA polymerase Zymo Taq™ premix (Zymo Research, USA), and the PCR system was as follow: 4 min at 95 °C, followed by 40 cycles of denaturation for 30 s at 95 °C, annealing for 30 s at 46 °C, extension for 20 s at 72 °C, and a final extension at 72 °C for 7 min. The PCR products were gel-purified and subcloned into pMD18-T vectors (TaKaRa, Japan) and the clones confirmed by PCR were selected for DNA sequencing (BGI, China). Bisulfite sequencing data and the C–T conversion rate were analyzed by BIQ Analyzer software. Methylation data from bisulfite sequencing were evaluated by computing the percentage of methylated CpGs of the total number of CpGs.

### Statistical analysis

The results of the BrdU, qRT-PCR and western assays were statistically analyzed using a two-tailed t-test analysis (Excel, Microsoft 2007). All data were collected from 3 independent experiments. P value of <0.05 was considered to be statistically significant difference, P value of <0.01 was considered to be highly significant difference and P value of <0.001 was considered to be extremely significant difference.

## Additional Information

**How to cite this article**: Zheng, L. *et al*. The Modification of Tet1 in Male Germline Stem Cells and Interact with PCNA, HDAC1 to promote their Self-renewal and Proliferation. *Sci. Rep.* **6**, 37414; doi: 10.1038/srep37414 (2016).

**Publisher's note**: Springer Nature remains neutral with regard to jurisdictional claims in published maps and institutional affiliations.

## Supplementary Material

Supplementary Tables and Figures

## Figures and Tables

**Figure 7:**