# Supplementary material for: The Modification of Tet1 in Male Germline Stem Cells and Interact with PCNA, HDAC1 to promote their Self-renewal and Proliferation
Source: Sci Rep. 2016 Nov 18;6:37414. doi: 10.1038/srep37414 (PMC5114665; doi:10.1038/srep37414)
Supplement: Supplementary Tables and Figures [file srep37414-s1.doc]

**The Modification of Tet1 in Male Germline Stem Cells and Interact with PCNA, HDAC1 to promote their Self-renewal and Proliferation**

Liming Zheng#1, Yuanxin Zhai#1, Na Li1, Fanglin Ma1, Haijing Zhu1, Xiaomin Du1, Guangpeng Li2, Jinlian Hua1^*^

1. College of Veterinary Medicine, Shaanxi Centre of Stem Cells Engineering

& Technology, Northwest A&F University, Yangling, Shaanxi, 712100 China

1. Key Laboratory for Mammalian Reproductive Biology and Biotechnology, Ministry of Education, Inner Mongolia University, Hohhot, 010021, China

# These authors contributed equally to this work.

^*^ Corresponding author.

*Correspondence to [jinlianhua@nwsuaf.edu.cn]

**Table S1-1 PCR Primers for gene clone**

| Gene | Primer sequence | Product (bp) | Tm (°C) |
| --- | --- | --- | --- |
| Pcna | CTAGCTAGCCTCAGGCGTTCGTAATCG | 958 | 62 |
|  | CGAGCTCCTGGCATCTCAGAAGCAG |  |  |
| Hdac1 | CTAGCTAGCATTCTTTCTGTTGTGGGAGT | 1500 | 65 |
|  | CGAGCTCCTGAAGGGTTGTGGGATA |  |  |
| Sin3A | CTAGCTAGCCCTGAGCATGAACGCAGAA | 3988 | 63 |
|  | TCCCCCGGGCACCAGCCACGCACAGAT |  |  |
| Oct4  Promoter | AAGGGATTTTTTATTTTTGTTGTGT | 275 | 62 |
|  | ACCCACTAACCTTAACCTCTAACC |  |  |

**Table S1-2 Restriction Enzyme cutting site of pIRES2-AcGFP**

| Gene | Upstream site | Downstream site |
| --- | --- | --- |
| Pcna | *Nhe*Ⅰ | *Sac*Ⅰ |
| Hdac1 | *Nhe*Ⅰ | *Sac*Ⅰ |
| Sin3A | *Nhe*Ⅰ | *Sma*Ⅰ |

**Table S1-3 Homology comparison of Tet1 conserved domains among cattle, goat, human and mouse**

| Gene | Conserved domain hits | Accession | Interval |
| --- | --- | --- | --- |
| Cattle | zf-CXXC | pfam02008 | 582-621 |
|  | 2OG-Fell-Oxy super family | cl21496 | 1617-1725  2019-2064 |
| Goat | zf-CXXC | pfam02008 | 582-621 |
|  | 2OG-Fell-Oxy super family | cl21496 | 2039-2084 |
| Human | zf-CXXC | pfam02008 | 585-624 |
|  | 2OG-Fell-Oxy super family | cl21496 | 2007-2052 |
| Mouse | zf-CXXC | pfam02008 | 568-607 |
|  | 2OG-Fell-Oxy super family | cl21496 | 1918-1963 |

**Table S2 The primers for qRT-PCR**

| Gene | Strand | Sequence | PCR Product (bp) | Tm (°C) |
| --- | --- | --- | --- | --- |
| Gapdh | F | CCACGCCATCACTGCCACCC | 116 | 60 |
|  | R | CAGCCTTGGCAGCGCCAGTA |  |  |
| Gfra1 | F | GGACAGGCAGCAGGAAATA | 200 | 60 |
|  | R | GTCTCCTGTCCCAGTCAAA |  |  |
| Plzf | F | CACCGCAACAGCCAGCACTAT | 127 | 59 |
|  | R | CAGCGTACAGCAGGTCATCCAG |  |  |
| Pcna | F | AGTGGAGAACTTGGAAATGGAA | 167 | 60 |
|  | R | GAGACAGTGGAGTGGCTTTTGT |  |  |
| Ccnd1 | F | TGAACTACCTGGACCGCT | 212 | 59 |
|  | R | CAGGTTCCACTTGAGYTTGT |  |  |
| Scp3 | F | GTATGGAGGACTTGGAGA | 138 | 60 |
|  | R | GAGACTTTCGGACACTTGC |  |  |
| Sin3A | F | GAGGCTGCTTCGGATTTGT | 105 | 60 |
|  | R | GCTGTCACTCTTGTCTCGCTTA |  |  |
| HoxB1 | F | CCTTCGCACCAACTTCACCA | 165 | 59 |
|  | R | CTCCCGCTTCTTCTGCTTCAT |  |  |
| mTet1 | F | GAGCCTGTTCCTCGATGTGG | 202 | 60 |
|  | R | CAAACCCACCTGAGGCTGTT |  |  |
| gTet1 | F | AAGGGAAGGAATGGAAGCCAAGATC | 108 | 60 |
|  | R | CCAGAACGAGGAATGGGTTGAGTAA |  |  |
| gTet3 | F | ACCCATCCTTTGCTCCTGACG | 205 | 60 |
|  | R | TCGGGCCGCTTGAATACTG |  |  |
| Oct4 | F | GTGTTCAGCCAAAAGACCATCT | 156 | 60 |
|  | R | GGCCTGCATGAGGGTTTCT |  |  |
| FoxO1 | F | CAACATCCGCAGTCAAT | 284 | 50 |
|  | R | GTCCATGAGGTCGTTCC |  |  |
| CasPas3 | F | CAGACAGTGGTGCTGAGGATGA | 208 | 60 |
|  | R | GCTACCTTTCGGTTAACCCGA |  |  |

**Supplementary Information**

**Fig 4**

**Fig 4D**

**
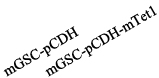

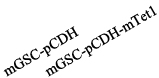
**


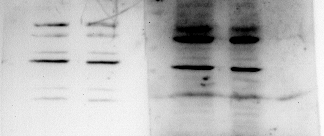


**p-ERK**

**ERK**


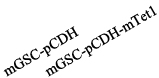


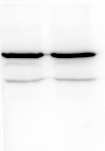


**ACTIN**

**Fig 4E**


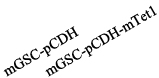

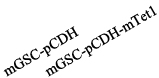


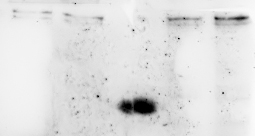


**JMJD3**

**EZH2**

**
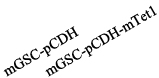
**

**
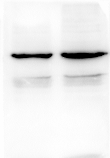
**

**ACTIN**

**Fig 4B**


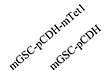


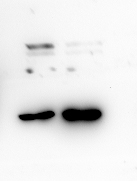


**ACTIN**

**SIN3A**

**Fig 5**

**Fig 5B**


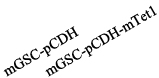

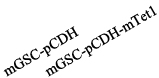

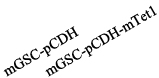


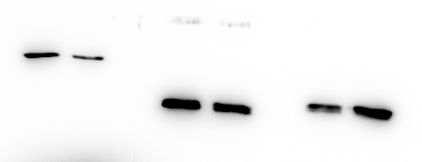


**ACTIN**

**S6**

**pS6**

**Fig 5C**


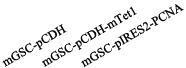


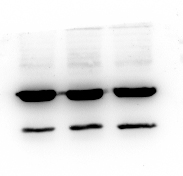


**ACTIN**

**PCNA**

**Fig 5E**


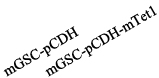


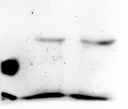


**ACTIN**

**HDAC1**

**Fig 5F**

In Fig 5F, its ACTIN is the same with Fig 5C, because this a same membrane, After exposure with ACTIN and PCNA in Fig 5C, we treated the membrane with antibody removing liquid and secondly incubated antibody HDAC1, after exposure, we got stripe of HDAC1.


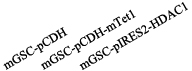


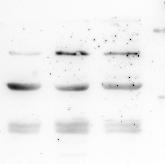


**HDAC1**

**Fig 5D**

**IgG Input Myc IP**


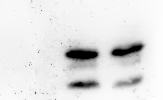


**PCNA IB**

The same membrane with more than double exposure with HDAC1, we choes the stripe with exposure for 60 second.

**Input Myc IP IgG Input Myc IP IgG**


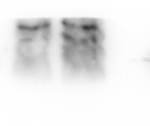

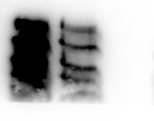


**HDAC1 IB**

**Exposure for**

**60 second**

**HDAC1 IB**

**Exposure for 5 second**

**Fig 6**

**Fig 6D**


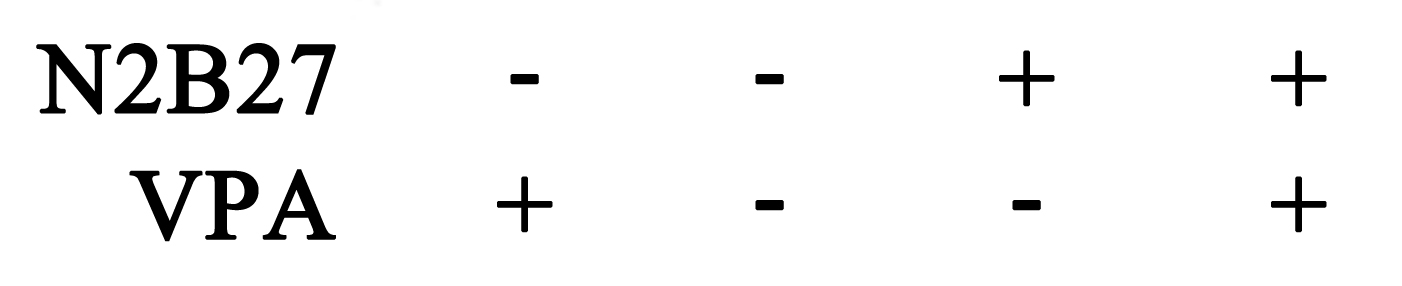


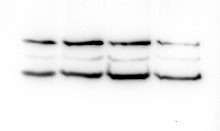


**HDAC1**

**ACTIN**

**Fig 6E**

The same membrane with more than double exposure with OCT4 and ACTIN, we choes the stripe of ACTIN with exposure for 1 second and OCT4 with exposure for 60 second.

**VPA- VPA+ VPA- VPA+ VPA- VPA+ VPA- VPA+**


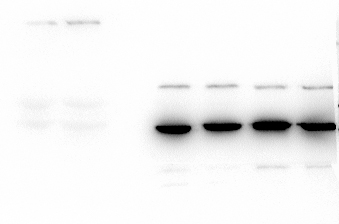

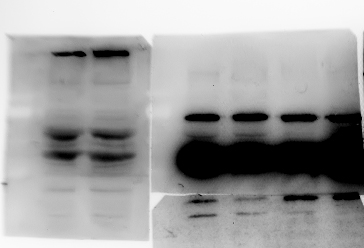


**OCT4**

**Exposure for 1 second**

**ACTIN**

**Exposure for 1 second**

**OCT4**

**Exposure for 60 second**

**ACTIN**

**Exposure for**

**60 second**
